# An overview of the host spectrum and distribution of Calodium hepaticum (syn. Capillaria hepatica): part 2—Mammalia (excluding Muroidea)

**DOI:** 10.1007/s00436-013-3692-9

**Published:** 2013-11-21

**Authors:** Hans-Peter Fuehrer

**Affiliations:** Institute of Parasitology, Department of Pathobiology, University of Veterinary Medicine Vienna, Veterinärplatz 1, 1210 Vienna, Austria

## Abstract

*Calodium hepaticum* (syn. *Capillaria hepatica*) is a globally distributed zoonotic nematode with low host specificity and a high affinity to the liver. Although murid rodents are the main definite hosts, various other mammals can be affected with hepatic capillariasis: non-murid rodents, Insectivora, Chiroptera, Lagomorpha, Artiodactyla, Perissodactyla, Hyracoidea, Marsupialia, Carnivora, and Primates. Overall, more than 180 mammalian species (including humans) are known as suitable hosts of this pathogen. This review gives an overview of the distribution and host spectrum of *C. hepaticum* in non-Muroidean mammals in wildlife and zoos as well as in domesticated and laboratory animals. Furthermore, the role of spurious infections in animals and the dissemination of *C. hepaticum* by mammalian and non-mammalian animals are summarized.

## Introduction

*Calodium hepaticum* is a worldwide-distributed zoonotic parasite with a high affinity to the liver. It is the causative agent of hepatic capillariasis and has low host specificity. This parasite is mainly diagnosed during liver biopsies or through necroscopy because the eggs of this nematode are only released into the environment after the host's death (after predation, cannibalism, or decay). The main hosts are rodents of the subfamilies Murinae and Arvicolinae. Although murids are the most important hosts, this parasite was documented in more than 70 non-murid species. This review focuses on the mammalian (excluding Muroidea) host spectrum and its geographic distribution in those hosts. Furthermore, the role of spurious infections in animals and the dissemination of *C. hepaticum* are summarized. Information about the pathogenesis, ecology, and host spectrum in Muriodea is given elsewhere (e.g., Fuehrer et al. 2011; Fuehrer 2013; Schmidt 2001).

For data evaluation, the systematic search was based on electronic databases (Scopus, PubMed, Google Scholar) and previous summaries (e.g., Schmidt 2001). The search terms *Capillaria hepatica*, *Calodium hepaticum*, *Hepaticola hepatica*, *Trichocephalus hepaticus*, and hepatic capillariasis were used. An attempt was made to include only those studies where the scientific names of the host and parasite were given clearly. Furthermore, spurious infections (= pseudoparasitism) were differentiated as far as possible from hepatic capillariasis.

## Taxonomy, morphology, and biology

*C. hepaticum* is a member of the family Capillaridae (Order: Trichocephalida). The parasite is also known under the synonym *Capillaria hepatica*. Although Moravec (1982) included this nematode in the genus *Calodium*, many scientists still use the synonym *C. hepatica*. Further synonyms are *Hepaticola hepatica* (Hall 1916) and *Trichocephalus hepaticus* (Bancroft, 1893) (Fuehrer et al. 2011).

The taxonomy of the family Capillaridae is pending. All species out of the former genus *Capillaria* are included in the family Capillaridae. A recent study has shown that the family Capillaridae seems to be monophyletic and can be clearly separated from Trichuridae (Guardone et al. 2013). Although most species parasitize in animals, three are known to also infect humans: *Paracapillaria philippinensis* (syn. *Capillaria philippinensis*), *Eucoleus aerophila* (syn. *Capillaria aerophila*), and *C. hepaticum* (syn. *C. hepatica*).

Adult *C. hepaticum* are long slender-shaped nematodes with a narrow anterior body part (0.007–0.01 mm). The posterior body part becomes gradually thicker. Sexual dimorphism is present (females 27–100 mm; males 15–50 mm) (reviewed in Schmidt 2001). The eggs resemble typical trichurid eggs but differ in size (40–67 × 27–35 μM). The eggs are barrel-shaped, striated, and with polar plugs. Numerous minipores are present on the outer shell. The four larval stages differ in size (reviewed in Schmidt 2001).

*C. hepaticum* has a high affinity to the liver and is the causative agent of hepatic capillariasis. The life cycle is a direct one. After the ingestion of embryonated eggs, L1 larvae hatch in the area of the caecum and invade the liver via the portal vein system. Adult *C. hepaticum* nematodes live in the liver parenchyma (life span 18–60 days) where females lay unembryonated eggs into the liver parenchmya. The eggs develop in the liver parenchyma to the eight-cell stage. After the death of the host (cannibalism, predation, decay), the eggs are released into the environment and embryonate (depending on the environmental conditions) to the infective stage. The cycle closes with the ingestion of embryonated eggs by a new host (reviewed in Schmidt 2001). The ingestion of unembryonated eggs leads to spurious infections (= pseudoparasitism) where the non-infective eggs are shed into the environment with the feces.

## Host spectrum

The main hosts of *C. hepaticum* are several murid rodent species with the highest prevalences in synanthropic Murinae (e.g., Norway rat). The parasite was documented in more than 90 Muroidean rodent species of the subfamilies Murinae, Deomyinae, Arvicolinae, Neotominae, Cricetinae, Sigmodontinae, Gerbillinae, and Cricetomyinae (Führer et al. 2010; Fuehrer 2013).

However, hepatic *C. hepaticum* infections were also found in Caviidae, Erethizontidae, Castoridae, Myocastoridae, Sciuridae, Geomyidae, Dipodidae, Nesomyidae, and Cuniculidae (Table 1). In wildlife, North American porcupines (USA; 9 % of 53), nutrias (Argentina; 3.6 % of 108), northern pocket gophers (USA; 39 % of 46), Brazilian guinea pigs (Peru; 6.9 % of 143), and lowland pacas (Brazil; 20 % of 5) were evaluated as suitable hosts of this parasite (Dittmar 2002; Hamir and Rupprecht 2000; Martino et al. 2012; Todd et al. 1971).

**Table 1:** *Calodium hepaticum* in non-Muroidea rodents

Classification	Species	Country/countries	References
Caviidae	Domestic guinea pig (*Cavia porcellus*)	Pets: Hungary	Meszaros and Varga (1976)
		Peru	Olortegui (1961); Gonzales (1970)
	Brazilian guinea pig (*Cavia aperea*)	Argentinia	Morini and Boero (1958)
		Peru	Dittmar (2002)
		Peru	Olortegui (1961); Gonzales (1970)
	*Cavia* sp.	Brazil	Mentioned in Dittmar (2002)
	Capybara (*Hydrochoerus hydrochaeris*)	Brazil (Nhecolândia)	Costa and Catto (1994)
Erethizontidae	North American porcupine (*Erethizon dorsatum*)	USA	Hamir and Rupprecht (2000)
Castoridae	North American beaver (*Castor canadensis*)	USA (National Zoological Park) District of Columbia	Chitwood BG (Chitwood 1934)
	Eurasian beaver (*Castor fiber*)	Hungary (Zoo)	Mészáros and Kemenes (1973)
		Former UDSSR	Pavlov (1955)
		Former UDSSR (several cases in a zoological park)	Mentioned in Mészáros and Kemenes (1973)
		Russia	Romašov (1996)
Myocastoridae	Nutria (*Myocastor coypus*)	Japan	Matsudate et al. (2003)
		Germany (Saxony)	Seidel (1954)
		Argentina	Vogelsang and Espin (1949)
		Argentina	Martino et al. (2012)
Sciuridae	Brazilian squirrel (*Sciurus aestuans*)	Brazil	Freitas and Lent (1936)
		Argentina	Vogelsang and Espin (1949)
	Caucasian squirrel (*Sciurus anomalus*)	Former UDSSR	Pavlov (1955)
	Fox squirrel (*Sciurus niger*)	USA (Louisiana)	McQuown (1954)
	Eurasian red squirrel (*Sciurus vulgaris*)	UK (north Wales)	Stidworthy et al. (2009)
	American red squirrel (*Tamiasciurus hudsonicus*)	Canada (lab infection experiment)	Freeman and Wright (1960)
	Korean squirrel (*Tanias sibericus*)	Spain pet shop (imported from China)	Carrasco et al. (2006)
	*Sciurus* sp.	Turkey	Merdivenci (1970)
	Cape ground squirrel (*Xerus inauris*)	South Africa (Eastern Free State)	Erlwanger et al. (2009)
	Richardson's ground squirrel (*Urocitellus richardsonii*)	USA (Montana)	Luttermoser (1938)
		Canada (Alberta)	Brown and Roy (1943)
	Domesticated squirrels	China	Mentioned in Wang et al. (2013)
	Black-tailed prairie dog (*Cynomys ludovicianus*)	USA	Weidman (1925)
		USA (zoo)	Landolfi et al. (2003)
		USA (Pennsylvania)	Doran (1955)
		England (zoo)	Redrobe and Patterson-Kane (2005)
	Alpine marmot (*Marmota marmota*)	Spain	Gortazar et al. (1994)
	Groundhog (*Marmota monax*)	USA	Reynolds and Gavutis Jr. (1975)
		USA (Pennsylvania)	Doran (1955)
		Germany (Lab groundhogs imported from the USA)	Hilken et al. (2003)
		France	Gevrey et al. (1996)
	Red-cheeked flying squirrel (*Hylopetes spadiceus*)	Malaysia	Liat et al. (1977)
Geomyidae	Plains pocket gopher (*Geomys bursarius*)	USA	Ubelaker and Downhower (1965)
	Northern pocket gopher (*Thomomys talpoides*)	Canada (Alberta)	Lubinsky (1956; 1957)
		USA (Wyoming)	Law and Kennedy (1932)
		USA (Wyoming)	Todd et al. (1971)
		USA	Rausch (1961)
Dipodidae	Woodland jumping mouse (*Napaeozapus insignis*)	Canada (Alonquin Park)	Freeman and Wright (1960)
Nesomyidae	Malagasy giant rat (*Hypogeomys antimena*)	England (zoo)	Redrobe and Patterson-Kane (2005)
Cuniculidae	Lowland paca (*Cuniculus paca*)	Brazil (Acre)	Almeida et al. (2012)
		Costa Rica	Matamoros et al. (1991)

Furthermore, *C. hepaticum* was documented in at least 69 species out of 25 families in non-rodent mammalian including Insectivora, Chiroptera, Lagomorpha, Artiodactyla, Perissodactyla, Hyracoidea, Marsupialia, Carnivora, and Primates (Table 2). In wildlife, hepatic capillariasis was documented in several studies: pronghorn antelopes (Canada; 4/41), red foxes (Italy; 1/75), crab-eating foxes (Brazil; 5.56 %), pampas foxes (Brazil; 13.64 %), and mountain gorillas (Rwanda; 10/19) (Barrett and Chalmers 1972; Graczyk et al. 1999; Macchioni et al. 2013; Ruas 2005). The true burden of this parasite in wildlife is not clear. Numerous documented cases of *C. hepaticum* in non-murid mammals were reported from zoological gardens and laboratories or in domesticated animals.

**Table 2:** *Calodium hepaticum* in other mammals other than rodents

Classification	Species	Country/countries	References
Insectivora			
Erinaceidae	European hedgehog (*Erinaceus europaeus*)	Switzerland	Brander et al. (1990, 1991)
		Turkey	Merdivenci (1970)
Soricidae	Smoky shrew (*Sorex fumeus*)	USA	Solomon and Handley (1971)
	Northern short-tailed shrew (*Blarina brevicauda*)	USA	Solomon and Handley (1971)
	Laxmann's shrew (*Sorex caecutiens*)	???	Mentioned in Tinnin et al. (2011)
	Long-tailed shrew (*Sorex dispar*)	USA	Solomon and Handley (1971)
	Cinereus shrew (*Sorex cinereus*)	USA	Solomon and Handley (1971)
	Common shrew (*Sorex araneus*)	Austria	Frank (1977)
	Shinto shrew (*Sorex shinto*)	Japan	Iwaki et al. (1993)
	Long-clawed shrew (*Sorex unguiculatus*)	Japan	Chabaud et al. (1963)
	Asian house shrew (*Suncus murinus*)	Indonesia	Brown et al. (1975a)
	Eurasian water shrew (*Neomys fodiens*)	England (Gloucestershire)	Stidworthy et al. (2009)
	Greater white-toothed shrew (*Crocidura russula*)	France—Mulhouse Zoo	Apéry (2012)
		France—Lyon Zoo	Apéry (2012)
Chiroptera			
Pteropodidae	Lesser short-nosed fruit bat (*Cynopterus brachyotis*)	Indonesia	Brown et al. (1974)
Lagomorpha			
Leporidae	European hare (*Lepus europaeus*)	???	Hall (1916)
		Germany (Saxony-Anhalt)	Haupt and Stubbe (1990)
		Germany (Saxony)	Schüppel (1980)
		Austria	Kutzer and Frey (1976)
		England	Nicoll (1911)
		Hungary	Sugár et al. (1978)
		Former CSSR	Zajíček (1958)
	Mountain hare (*Lepus timidus*)	Georgia	Kankava et al. (1971)
		Austria (zoo)	Eder 2008
	European rabbit (*Oryctolagus cuniculus*)	Switzerland	Brander et al. (1991); Hörning (1974)
		England	Morgan (1931)
	Domestic rabbit (*Oryctolagus cuniculus forma domestica*)	France	Gevrey and Chirol (1978)
	New Zealand white rabbits	UK (rabbits from commercial distributor)	Mowat et al. (2009)
	Eastern cottontail (*Sylvilagus floridanus*)	USA	Layne (1970); Layne and Winegarner (1971)
Ochotonidae	Plateau pika (*Ochotona curzoniae*)	China (Gansu)	In Wang et al. (2013)
Artiodactyla			
Antilocapridae	Pronghorn antelope (*Antilocapra americana*)	Canada (Alberta)	Barrett and Chalmers (1972)
Tayassuidae	Collared peccary (*Pecari tajacu*)	Brazil	Mandorino and Rebouças (1991)
	White-lipped peccary (*Tayassu pecari*)	Panama	Foster and Johnson (1939)
		Brazil	Soares et al. (2011)
	*T. pecari* or *P. tajacu*	Brazil (Amazonas)	Gonçalves et al. (2012)
Bovidae	Kirk's dik-dik (*Madoqua kirkii*)	USA (zoo)	Partington and Montali (1986)
	Cattle (*Bos primigenius*)	Japan (Hokkaido)	Nakamura (2005)
Suidae	Domestic pig (*Sus scrofa domesticus*)	China	Zhang (1990)
Perissodactyla			
Equidae	Horse (*Equus ferus caballus*)	Canada	Nation and Dies (1978)
		England	Munroe (1984)
Tapiridae	Brazilian tapir (*Tapirus terrestris*)	Brazil (Parana)	Mangini et al. (2002)
Hyracoidea			
Procaviidae	Southern tree hyrax (*Dendrohyrax arboreus*)	Democratic Republic of the Congo	Fain (1953)
Marsupialia			
Didelphidae	Big-eared opossum (*Didelphis aurita*)	Paraguay	Canese (1973)
	Common opossum (*Didelphis marsupialis*)	Columbia	CIAT (1973)
Macropodidae	Agile wallaby (*Macropus agilis*)	Australia	Canfield and Hartley (1992)
	Parma wallaby (*Macropus parma*)	Australia	Canfield and Hartley (1992)
	Red kangaroo (*Macropus rufus*)	Australia	Canfield and Hartley (1992)
Potoroidae	Rufous rat-kangaroo (*Aepyprymnus rufescens*)	Australia	Canfield and Hartley (1992)
	Woylie (*Bettongia penicillata*)	Australia	Canfield and Hartley (1992)
		??? Zoo	Mentioned in Redrobe and Patterson (2005)
	Potoroideae spp.	Germany	Schmidt (1975)
Carnivora			
Mephitidae	Eastern spotted skunk (*Spilogale putorius*)	USA	Layne and Winegarner (1971)
Canidae	Domestic dog (*Canis lupus familaris*)	New Zealand	Anon. (1982)
	West highland white terrier cross	Great Britain	Lloyd et al. (2002)
		Italy	Carta (1939)
		Switzerland	Brander et al. (1990, 1991)
		USA (Washington)	Wright (1930)
		India	Rao et al. (1975)
		Brazil	Saliba et al. (1965); Santos and Barros (1973); Silveira et al. (1975)
		Brazil	Ilha and Barros (Ilha MR da S, Barros CSL de 2000)
		Brazil	Palma et al. (2009)
		South Africa	Smit (1960)
		Nigeria	Ajayi et al. (2011)
		Australia	Stokes (1973)
	Gray wolf (*Canis lupus*)	Russia	Romašov (1996)
	Coyote (*Canis latrans*)	Canada	Wobeser and Rock (1973)
		USA	Crowell et al. (1978); Custer and Pence (1981)
	Red fox (*Vulpes vulpes*)	Italy	Macchioni et al. (2013)
	Crab-eating fox (*Cerdocyon thous*)	Brazil	Ruas (2005)
	Pampas fox (*Lycalopex gymnocercus*)	Brazil	Ruas (2005)
	Maned wolf (*Chrysocyon brachyurus*)	Brazil	Curial (1954)
Felidae	Domestic cat (*Felis catus*)	Brazil	Santos and Barros (1973)
		Brazil	Ilha and Barros (2000)
		Slovakia	Mituch (1968)
		Nigeria	Okaeme (1985, 1986)
	Cougar (*Puma concolor*)	Brazil	Quadros et al. (2009)
Primates			
Lemuridae	Ring-tailed lemur (*Lemur catta*)	Chile (zoo)	Zordan et al. (2012)
		??? Zoo	Mentioned in Redrobe and Patterson (2005)
Cercopithecidae	Vervet monkey (*Chlorocebus pygerythrus*)	South Africa	Fripp and Kaschula (1974)
	Grivet (*Chlorocebus aethiops*)	South Africa	Fripp and Kaschula (1974)
	Rhesus macaque (*Macaca mulatta*)		Brack (1987)
	Crab-eating macaque (*Macaca fascicularis*)		Brack (1987)
		UK (wild-caught laboratory-maintained primates)	Abbott and Majeed (1984)
	Celebes crested macaque (*Macaca nigra*)	England (zoo)	Pizzi et al. (2008)
		England (zoo)	Stidworthy et al. (2009)
	Tibetan macaque (*Macaca thibetana*)	China (Huangshan mountain of Anhui)	Mentioned in Wang et al. (2013)
	Northern plains gray langur (*Semnopithecus entellus*)	Belgium/Sri Lanka	Kumar et al. (1983)
	Gelada (*Theropithecus gelada*)	USA (zoo)	Jensen and Huntress (1982)
Atelidae	Mexican spider monkey (*Ateles geoffroyi velerosus*)	Mexico (Chiapas)	Caballero and Grocott (1952)
	Geoffroy's spider monkey (*Ateles geoffroyi*)	Panama	Foster and Johnson (1939)
	Red-faced spider monkey (*Ateles paniscus*)	Brazil	Soares et al. (2011)
	Humboldt's woolly monkey (*Lagothrix lagotricha*)		Brack (1987)
Pitheciidae	White-faced saki (*Pithecia pithecia*)	England (zoo)	Pizzi et al. (2008)
	Red bald-headed uakari (*Cacajao calvus rubicundus*)		Brack (1987)
Cebidae	White-headed capuchin (*Cebus capucinus*)	Panama	Foster and Johnson (1939)
Hominidae	Human (*Homo sapiens*)	Worldwide	Reviewed in Fuehrer et al. (2011)
	Mountain gorilla (*Gorilla beringei beringei*)	Rwanda (Parc National de Volcans)	Graczyk et al. (1999)
	Western gorilla (*Gorilla gorilla*)	Poland (spurious infection/zoo)	Paciepnik (1976)
	Common chimpanzee (*Pan troglodytes*)	???	de Gasperi (1913)
		Free ranging	Troisier et al. (1928)
		USA (lab animals originated from West Africa)	Sadun et al. (1970)
Callitrichidae	Pied tamarin (*Saguinus bicolor*)	UK (zoo)	Stidworthy et al. (2009)
		France—Mulhouse Zoo	Apéry (2012)
		Portugal—Lisbon Zoo	Correia et al. (2011)
	Red-handed tamarin (*Saguinus midas*)	Portugal—Lisbon Zoo	Correia et al. (2011)
	Goeldi's monkey (*Callimico goeldii*)	Portugal—Lisbon Zoo	Correia et al. (2011)
	White-headed marmoset (*Callithrix geoffroyi*)	1 case each in 3 zoos UK (zoo)	Stidworthy et al. (2009)
		Spain	Fernández-Bellon et al. (2001)

## Zoos

Several hepatic cases with *C. hepaticum* had been observed in zoological gardens. Various studies documented single cases of this parasite. In some reports, more than one animal of a single species were infected: black-tailed prairie dogs (USA; 5/21; UK; 45 % of 20) and Kirk's dik-diks (USA; 7/18) (Landolfi et al. 2003; Partington and Montali 1986; Redrobe and Patterson-Kane 2005). Most of the cases in primates were found in zoos.

Several studies tried to analyze the relationships of commensal rodents (e.g., Norway rats, house mice) to infections of animals in zoos (e.g., Juncker-Voss et al. 2000). In zoos, high prevalences of free-ranging rats, mice, and shrews were observed: Norway rats (Baltimore Zoo, USA, 75 % of 845; Lisbon Zoo, Portugal 42 % of 50), house mice (Vienna Zoo, Austria, 42.7 % of 166; Lisbon Zoo, Portugal 22 % of 50), and greater white-toothed shrews (France; 10–25 %) (Apéry 2012; Crespo 2012; Farhang-Azad 1977; Juncker-Voss et al. 2000).

## Laboratory animals and pet shops

*C. hepaticum* was found in various pet shops and laboratories, for example, in one out of four Korean squirrels imported from China to Spain, 3 out of 155 lab groundhogs imported from the USA to Germany, 13 out of 160 New Zealand White rabbits in France from a commercial distributor, two cases in common chimpanzees which were lab animals originating from West Africa, and 0.6 % of 472 wild-caught laboratory-maintained crab-eating macaques (Abbott and Majeed 1984; Carrasco et al. 2006; Hilken et al. 2003; Mowat et al. 2009; Sadun et al. 1970). It can be hypothesized that animals ingested embryonated eggs while living wild and/or with contaminated food.

## Domesticated animals

*C. hepaticum* was documented in domesticated mammal species like laboratory Norway rats, rabbits, cattle, pigs, horses, dogs, cats, domesticated guinea pigs, and squirrels. In Japan, hepatic capillariasis was observed in 2.25 % of 400 cattle, but the author did not classify the nematode as *C. hepaticum* because the pathogen was not reported in cattle before (Nakamura 2005). Furthermore, Ilha and Barros (2000) found *C. hepaticum* in the livers of 0.23 % of 3,927 dogs and 1.38 % of 435 cats examined in Brazil.

## Dispersal by animals and spurious infections

With the death of the animal host (cannibalism, predation, or decay), the eggs of *C. hepaticum* are released into the environment. The dissemination of eggs by ground beetles and rain worms had been reported, but their role in the importance of maintaining the life cycle of this parasite is unclear (Mobedi and Arfaa 1971; Schmidt 2001).

In humans, spurious infections are associated with the consumption of unembryonated eggs in soil or infected game (Fuehrer et al. 2011). The same can be considered for other carnivore and omnivore animals (Reperant and Deplazes 2005). Gonzalves et al. (2012) described the first case of a spurious infection in a dog in Brazil (Amazonas) after the dog was fed with raw game meat. In the Zoological Garden of Vienna, eggs of *C. hepaticum* were found in the feces of a Pallas's cat (*Otocolobus manul*) (Basso et al. 2005). Spurious infections have also been observed in Norway rats (6 %) and black rats (20 %), where cannibalism might be the mode of intake of unembryonated eggs (Firlotte 1948; Promkerd et al. 2008). In Madagascar, eggs from *Capillaria* sp. with the shape of *C. hepaticum* have been found in the feces of gray mouse lemurs (*Microcebus murinus*), greater hedgehog tenrecs (*Setifer setosus*), and black rats (*R. rattus*).

Furthermore, eggs of *C. hepaticum* were found in the feces of non-mammalian animals. In Malaysia, 2.83 % of large-billed crows shed eggs of this parasite with the feces (Lee et al. 2008). Eggs of *C. hepaticum* were also documented in fecal samples from reptiles fed with infected rodents (Pantchev and Tappe 2011). In an analysis of the intestinal content of two timber rattlesnakes (*Crotatus horridus*), eggs of *C. hepaticum* were documented (Solomo 1974).

Although many authors described spurious infections in animals, care should be taken to exclude mix-ups with other Capillaridae or Trichuridae shedding eggs of resembling morphology (e.g., Bork-Mimm and Rinder 2011; Di Cesare et al. 2011; Stuart et al. 2013; Traversa et al. 2011). With the absence of specific molecular diagnostic tools, the classification of *C. hepaticum* in spurious infections is based on the morphology of the eggs only. Consequently, the role of spurious infections for the maintenance of the life cycle of this nematode remains unclear.

## Conclusions

*C. hepaticum* is a worldwide-distributed zoonotic parasite with a high affinity to the liver and low host specificity. The main definite hosts are Murinae and Arvicolinae, but it has also been found in various other mammals of different families. Eggs are released into the environment after the death of the host only (decay, cannibalism, and predation). It is unclear which method of egg dispersal is the most effective one but it can be hypothesized that:

- Cannibalism is the most effective method of transmission in the case of rodents with a tendency to cannibalism and egg shedding in the burrow.
- Dispersal of unembryonated eggs by egg-shedding in feces (after cannibalism, predation by omnivores and carnivores, scavengers, dissemination by insects and earth worms) leads to the most infections in other mammals after the embryonation of the eggs.

Diagnosis is now based on liver biopsy and necroscopy, and it can be suggested that the true burden of this parasite is underrepresented. Novel molecular diagnostic tools are needed to allow species determination in cases of hepatic capillariasis and spurious infections.
